# Screen Use During Meals Among Young Children: Exploration of Associated Variables

**DOI:** 10.3390/medicina55100688

**Published:** 2019-10-14

**Authors:** Roma Jusienė, Vaidotas Urbonas, Ilona Laurinaitytė, Lauryna Rakickienė, Rima Breidokienė, Monika Kuzminskaitė, Rūta Praninskienė

**Affiliations:** 1 Institute of Psychology, Faculty of Philosophy, Vilnius University, LT-01513 Vilnius, Lithuania; vaidotas.urbonas@mf.vu.lt (V.U.); ilona.laurinaityte@fsf.vu.lt (I.L.); lauryna.rakickiene@fsf.vu.lt (L.R.); r.breidokiene@gmail.com (R.B.); monika.kuzminskaite@gmail.com (M.K.); ruta.praninskiene@santa.lt (R.P.); 2 Clinic of Children’s Diseases, Institute of Clinical Medicine, Faculty of Medicine, Vilnius University, LT-08406 Vilnius, Lithuania; 3 Pediatric Neurology Department, Children’s Hospital, Affiliate of Vilnius University Hospital Santaros Clinics, LT-08406 Vilnius, Lithuania

**Keywords:** screen time, children, eating habits, emotional and behavioral problems, body mass index

## Abstract

*Background and Objectives:* There is evidence that eating meals or snacks while watching TV is an obesogenic factor. Moreover, the patterns of TV and other screen use during meals begin early and persist. However, there are only a few studies to date which address the prevalence and predictors of young children’s exposure to screen during mealtimes. Thus, the present study aimed to investigate the prevalence and the associated factors of screen use during meals in early childhood. *Materials and Methods:* A cross sectional survey was conducted in Lithuania. Data of 847 children aged 2 to 5 years old (51.5% boys) were analyzed in this study. Parents completed the Child Behavior Checklist (CBCL/1½-5) and reported their children’s daily screen time, exposure to background TV, screen use during child’s meals, child and parental height and weight, and sociodemographic data. *Results:* More than half of children were exposed to screen during meals: 33.7% occasionally, several times per week or per month, and 22%—daily or during every meal. Overall daily screen time, background TV, consumption of junk food, child age, and emotional and behavioral problems were related to mealtime screen use (all associations significant at *p* < 0.01). Longer daily screen time (OR 1.01; 95% CI 1.00–1.01), more background TV (OR 1.26; 95% CI 1.10–1.45), and elder child age (OR 1.02; 95% CI 1.00–1.03) were significant predictors of occasional use of screen during meals. Also, longer daily screen time (OR 0.99; 95% CI 0.98–0.99), background TV (OR 0.78; 95% CI 0.66–0.91) together with no siblings’ status of a child (OR 0.42; 95% CI 0.25–0.69) increased the probability that children were fed in front of screens daily. *Conclusions*: This study confirmed the unfavorable associations among screen use during meals, daily screen time and junk food consumption in early childhood. In addition, first-time parents should get particular health providers’ attention as they are more likely to use screens during child’s mealtime.

## 1. Introduction

Young children are increasingly exposed to multiple screens [1,2,3,4,5,6,7] despite the continuous health recommendations that daily screen time for children aged 2–5 years should be less than 1 h [1,2,3,4,6,7,8,9,10]. The widespread and extensive use of various screen-based electronic media is influencing behavioral models, e.g., sedentary behavior [4,9,11,12,13,14], everyday eating [12,15,16,17,18,19] and sleeping behaviors [18,20,21], as well as general health and psychological wellbeing [5,13,14,22] in children of all age groups. The association between media exposure and childhood obesity has been supported by research over the past several decades [6,16,23], with both media exposure and obesity more prevalent among minorities and lower socioeconomic groups [24,25,26,27]. There is no association between child’s body mass index and screen media use in young children aged 0 to 5 years old [3,24], or the association is weak [4,28], although screen time in early childhood can predict obesity in later childhood [16,23]. The data suggest that screen time leads to obesity through increased eating while viewing [12,16,18,29] and increased choices and/or exposure to low-nutrient calorie-dense foods [15,17,18,19,26]. Despite evidence that eating meals or snacks while watching TV is an obesogenic behavior [9,16], and the patterns of TV and other screen use during meals begin early and persist [26,27], there are only a few studies to date which address the prevalence [26] and predictors of young children’s eating with screens [26,27,28]. Thimming et al. (2017) reported that 65% of the children in their sample were exposed to TV during meals for at least one time point within the first 2 years of life, and more than one-third were reported to be exposed to TV during meals at each surveyed time point from ages 0 to 4 years [26].

Screen use during meals can be more prevalent in young children due to increased overall screen time and background TV, especially because of positive parental attitudes toward screens and shared family environments, where screens are used extensively by other family members [3,8,25,27,30,31,32,33,34]. On the other hand, behaviors of infants and toddlers can be directly influenced by their parents’ or other caretakers’ efforts to nurture and regulate them [15,17,35]. Thus, screens during meals can be used as a parental aid to feed the underweight child and/or child who does not eat well (e.g., refuses food) [18]. In addition, toddlers with difficult behaviors or self-regulation problems may be placed in front of screen by their caregivers more frequently and during meals [27,36]. Studies examining toddler and preschooler mealtime screen use are limited [16,18]. Mealtime screen use in this age group (2–5 years) is of particular interest as this is a period of rapid development of autonomous eating habits [18] and self-regulatory skills [36] with concurrent dependence on parental control and regulation of access to screens. Moreover, there are few studies on using various screens (not limited to TV) during mealtime in early childhood [3,4,26,27]. To evaluate background TV or mobile media is also important, as they are highly prevalent in children environments [36].

The aim of our study was to investigate the prevalence of screen use during meals as well as its associations with child’s overall screen time (defined as the amount of time spent using a screen-based media device such as television, computer, tablet, smartphone, or video game console) and background TV, child’s body mass index (BMI), emotional and behavioral problems, parental BMI and parental education in the population based on a normative sample of toddlers and preschoolers (children aged 2 to 5 years old).

## 2. Materials and Methods

### 2.1. Participants

The present research used cross-sectional data of participants of ongoing longitudinal study on electronic media use and young children’s health (for more information about study see also [22]). The inclusion criteria for participation in the study were to be a Lithuanian speaking parent or main caregiver of a child aged 1.5 to 5 years old. Parents responded to questions about themselves, their family environments, the target child data, and also filled in the Child Behavior Checklist (CBCL/1½-5) [37].

Data of 847 children aged 24 to 71 months (mean age 45.50 months, SD = 13.77; 48.5% girls and 51.5% boys) were used in this research. Most questionnaires (93.9%) were filled out by mothers. Mean age of respondent parents or caregivers was 33.37 years (SD = 5.15). The studied sample reasonably represents main characteristics of the families with children of similar age within the national population (for more detailed description of the sample see also Table 1).

### 2.2. Procedure

All respondents (parents or caregivers of children) were informed about the objectives of the study and signed the informed consent. The participants (parents or caregivers) were given a choice of paper or online alternative (74.4% and 25.6% of the sample chose these options, respectively) to be filled out at the location of their choice. Data for this research were collected from May to December 2017. The study was approved by Vilnius Regional Committee of Biomedical Research Ethics (permission no. 158200-17-906-417, issued on 11.04.2017).

### 2.3. Measures

Screen use during meals was measured on a 5 item scale, ranging from 1 (“never or almost never”) to 5 (“during every meal or always”) with a question on how often a child is exposed to screen (TV, tablet, smartphone, PC or other) while being fed by a parent or eating together with him/her.

Daily screen time was measured by one item on a scale of 1 (“almost no usage”) to 7 (“more than 4 h per day”) with separate questions for workdays and weekends. Parents were asked to include child’s time spent using TV, PC, tablet, smartphone or gaming station when estimating overall screen time. To assess the average child’s daily usage of screen, first each option was converted to minutes as follows: (1)—0 min, (2)—22.5 min, (3)—45 min, (4)—90 min, (5)—150 min, (6)—210 min, and (7)—270 min. Next, the following formula was used to evaluate daily screen time: (Screen time on workdays × 5 days + Screen time on weekends × 2 days)/7 days.

Background TV usage was measured on a scale from 1 (“almost never”) to 5 (“almost always”) with a question on how often the TV is switched on at child’s home even if nobody is watching it.

Frequency of child’s consumption of junk food was measured on a scale of 1 (“almost never or very rarely”) to 5 (“very often or almost daily”) for five types of junk food (low-nutrient calorie-dense food): chips, sweet beverages, sweets, bakery products, fast foods, and a cumulative score for the frequency of eating all kinds of junk food was calculated. The internal consistency of the scale measuring child’s consumption of junk food was sufficient for statistical analysis (Cronbach *α* = 0.59).

Emotional and behavioral problems were measured using Lithuanian version of the CBCL/1½-5 [37,38]. Parents reported how well each item described the behavior of their child during the last 2 months, using a 3-point scale (ranging from 0 to 2). The sum score of total problems (e.g., anxious/depressed, emotionally reactive, withdrawn, somatic complaints, attention problems, aggressive behavior, sleep problems) was used in this study. The scale was standardized for Lithuanian population and has a good internal consistency measure (Cronbach *α* = 0.93 for total problems) [38].

Child body mass index was calculated from the child height and body weight data as reported by parents, by dividing weight in kilograms by squared height in meters.

Parental *BMI* was calculated from the reported height and weight data of biological mother and father, by dividing weight in kilograms by squared height in meters.

Maternal and paternal education. Each parent’s educational level was assigned a value from 1 (incomplete secondary education) to 5 (university degree), also including secondary, vocational and college education (see Table 1).

Child’s sibling status was assessed by dichotomous question on whether a child has sibling(s) or whether he/she is the only child.

### 2.4. Statistical Analysis

The SPSS 23.0 software package was used to analyze the data. Distribution of variables in groups was calculated using frequency distribution reports. Relationships between the studied variables were calculated using Spearman’s correlations. Comparisons of means among groups were conducted with the Mann-Whitney test (2 independent groups) or Kruskal Wallis test (more than 2 independent groups), and the Chi square test was used to evaluate differences between categorical or dichotomous variables. Multinomial logistic regression was used to predict categorical placement in or the probability of category membership on a dependent variable—screen use during meals—based on multiple independent variables. The dependent variable (screen use during meals) was transformed into three categories and coded as follows: 1—“never” (e.g., never or almost never), 2—“sometimes” (e.g., several times per month or two to three times per week), 3—“daily” (e.g., almost daily or during every meal).

Tests to see if the data met the assumption of collinearity (VIF value lower than 10, or the Tolerance higher than 0.1) indicated that multicollinearity was not a concern (Daily screen use, Tolerance = 0.81, VIF = 1.24; Background *TV*, Tolerance = 0.80, VIF = 1.26; *CBCL* total, Tolerance = 0.95, VIF = 1.06; Sibling status, Tolerance = 0.95, VIF = 1.05; Child age, Tolerance = 0.92, VIF = 1.09; Paternal *BMI*, Tolerance = 0.97, VIF = 1.04; Child BMI, Tolerance = 0.95, VIF = 1.05; Maternal education, Tolerance = 0.92, VIF = 1.09).

## 3. Results

Sociodemographic characteristics of the participants are presented in the Table 1. The majority of the participant children attended kindergarten (94.5%) and were living with the married parents (84.3%). Twenty-eight percent of children had no sibling. Of the mothers, 68.6% had university level of education. Of the fathers, 64.8% had university level of education.

**Figure 1:**
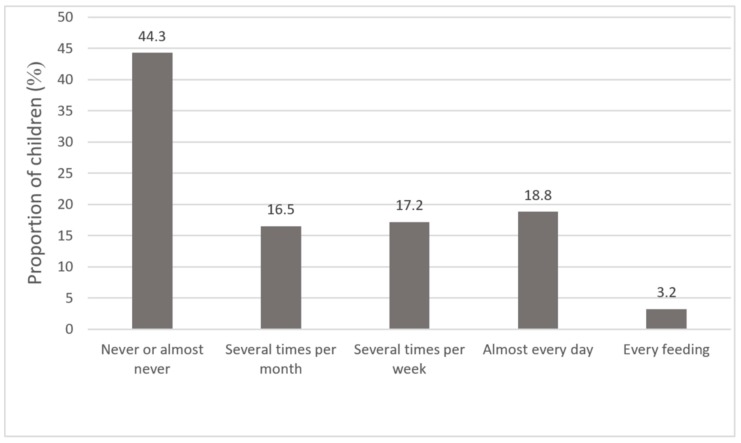
Distribution of children according to using screen during meals (N = 847).

Analysis of the prevalence of screen use during meals revealed that less than half of the children (44.3%) never or almost never were being fed using screen, 33.7% were reported sometimes to use screen during meals, and 22% of children were being fed using screen daily or during every meal (for more detailed reports see Figure 1). There were no significant sex differences according to the mealtime screen usage (χ^2^ = 0.39, *p* = 0.823). The mealtime screen usage was not related to kindergarten attendance (χ^2^ = 4.26, *p* = 0.372), family marital status (χ^2^ = 6.40, *p* = 0.380), and sibling status (χ^2^ = 5.83, *p* = 0.054).

Further we evaluated general amount of time children spend in front of the screens (see Table 1). Half of the parents comply with the recommendations for young children not to exceed one hour of daily screen time: 51.6% of the children were reported to spend on average less than 1 h per day using any type of electronic media device with screen. However, the remaining 48.4% of children exceeded the recommended 1 h daily of screen use. No differences of daily screen time were found among the only children and children who have at least one sibling (χ^2^ = 2.30, *p* = 0.129). Daily screen time was not related to child sex (Mann Whitney U = 84666.00, *p* = 0.919), family marital status (χ^2^ = 3.12, *p* = 0.210) and kindergarten attendance (χ^2^ = 2.37, *p* = 0.124).

Frequency of eating junk food was related to the sibling status and kindergarten attendance, e.g., children with siblings and those enrolled in kindergarten were reported to consume junk food more often (χ^2^ = 16.65, *p* < 0.000 and χ^2^ = 3.89, *p* = 0.049, respectively). However, it was not significantly associated with child sex (Mann Whitney U = 89597.00, *p* = 1.000) and family marital status (χ^2^ = 2.32, *p* = 0.313).

The correlational analysis of the study variables showed that the age of a child, average screen time per day, exposure to background TV, consumption of junk food, paternal BMI, total CBCL score were positively related to screen use during meals (see Table 2). Child being fed in front of screen was not related to maternal and paternal education, and maternal BMI. Maternal and paternal education was negatively related to daily screen time, exposure to background TV and total CBCL score. Moreover, the children’s BMI was related to the maternal BMI (but not paternal BMI), and it was significantly associated with lower maternal and paternal education, and younger child age. The consumption of junk food was positively related to screen use during meals and daily screen time, exposure to background TV, to BMI of both parents, total CBCL score and child age.

In the final stage of analysis, multinomial logistic regression was performed for the screen use during meals as a categorical outcome variable. In multivariate analysis we included the independent variables that reached significance in bivariate correlational analysis: Daily screen use, exposure to background TV, total CBCL score, sibling status, paternal BMI. In order to adjust for the child’s age and child’s BMI, and maternal education, we included these variables in multivariate analysis as well. Consumption of junk food was not included in the model based on the assumption that although eating junk food and eating / feeding in front of a screen may often occur together, it is highly unlikely that consumption of junk food is a precursor contributing to patterns of screen use during meals.

The results presented in Table 3 show that daily screen time (OR 0.99; 95% CI 0.98–0.99), exposure to background TV (OR 0.78; 95% CI 0.66–0.91) and no siblings status (OR 0.42; 95% CI 0.25–0.69) increased the probability that children daily used screen during meals as opposed to those who never used screen during meals. Daily screen time (OR 1.00; 95% CI 1.00–1.01) and exposure to background TV (OR 1.26; 95% CI 1.10–1.45), as well as elder child age (OR 1.02; 95% CI 1.00–1.03) increased the probability that children used screens sometimes during meals as opposed to those who never or almost never used screen while eating or being fed. Finally, longer screen time (OR 1.01; 95% CI 1.00–1.01) and no siblings status (OR 1.70; 95% CI 1.04–2.79) significantly predicted children being fed daily in front of screens as opposed to those, who were fed only sometimes (see Table 3).

## 4. Discussion

More than half (55.7%) of children aged 2 to 5 years old in the Lithuanian population based sample were exposed during meals to TVs or other screens at least occasionally. Twenty-two percent of the sample were fed in front of screen very often, e.g., daily or almost during every meal. Despite the claims that screen use during meals could relate to unhealthy eating behaviors [15,16,18], there are only a few studies which provide any data on the prevalence of TV exposure during meals in early childhood. To compare, one-third of children aged 0 to 2 years [27] and 0 to 4 years [26] were found to be fed in front of TV. Moreover, 65% of infants aged 0 to 2 in the U.S. were exposed to TV during meals at some time points [26] and 64% of children aged 6 months to 6 years in Australia watched TV during one or fewer mealtimes per day [32].

Nearly half of parents in our study could be considered as those not following the recommended screen time guidelines established in most post industrialized countries [3,7,9,11]. In our sample, 48.4% of children aged 2 to 5 years old were exposed to screens for more than one hour per day. Several recent studies [4,12] provide even larger proportions of children whose daily screen time exceeds recommended one. For example, 22% of Canadian children aged 3 to 4 years, as reported by Poitras et al. [4], and 15.8% of Canadian children aged 1 to 4 years as well as 54.5% aged 5 to 8 years old, as reported by Pyper et al. [12], were meeting the screen time guidelines. The other recent studies [1,2,39] also report high rates of children aged 0 to 5 years being exposed to mobile devices and electronic media screens.

The results of our study revealed that screen use during meals in early childhood is related to overall screen time and the use of TV as a background, and also associated with more frequent consumption of junk foods. These associations were also established in several other studies and reviews [3,4,25,27,32]. Not surprisingly, overall screen time as well as consumption of junk food also increased with child age in our sample. Contrary to another study [27], the results of our investigation also revealed that screen use during meals tend to be even more frequent as the children’s age increases. However, there is a lack of researches which provide clear data on the prevalence of screen use in various age groups of children. Similar to several other studies [3,24] we did not find that using a screen during meals was linked to young children’s BMI.

Parental education is not related to more frequent screen use during child’s meals and/or providing a child with junk food in our study. These findings contradict the results of numerous studies [23,24,25,26,27,32] claiming that parents with lower education (usually also representing less advantaged socioeconomic status) should be important targets for children’s health promotion [18,32]. On the other hand, lower parental education is related to longer daily screen time and background TV in our study, and this is in line with most of other studies [1,2,3,4,18,26,27]. The exposure to background TV and daily screen time are significant predictors of screen use during child’s meals. Thus, health care professionals should provide less educated parents with support and guidance aiming to limit screen use in child environment.

Two-parent household and child’s enrollment in kindergarten are not associated with screen use during child’s meals at home and with the overall screen time. The latter findings are in line with the results of systemic review [39]. Sibling status of a child (e.g., no siblings vs. having at least one sibling) was not related to daily screen time, and this is also in line with the mentioned review [39]. Although it is worth mentioning that based on results of regression analyses the only children (no sibling status) in our study were at higher odds of being fed more often in front of screen.

Finally, we revealed important correlations among children’s behavioral problems and unhealthy behaviors. Children who had higher scores of emotional and behavioral problems were also reported with higher exposure to background TV and longer daily screen time, and were more likely to be fed in front of screens. The adverse associations of fuzzy or difficult behaviors and higher exposure to screens were also observed in several studies with young children [4,27,36].

Some limitations of our study should be mentioned. First, as this study is cross-sectional, we cannot yet conclusively determine the directional effects. For example, whether parents are using screen to try to calm and entertain children with behavior problems, or whether overall media exposure as well as screen usage during meals, especially if apparent together with some other adverse factors (e.g., ineffective parenting or consumption of junk foods), can add to behavioral problems of children. Secondly, the child and parental BMI, time spent in front of screens, and screen use during meals in our study were reported by parents, thus these data could be not precise (e.g., while reporting weight and height or screen time) and/or biased (e.g., presenting more socially desirable answers regarding screen use during meals). Diary methods for tracking screen use and observational studies of children’s and parents’ behavior, as well as objectively measured weight and height should be used as more reliable and informative measures in future research.

Despite these limitations, the present study adds new data on screen time and young children’s nutrition-related health in two important aspects. First, we confirmed the unfavorable associations among screen use during meals, daily screen time and junk food consumption in early childhood. Secondly, the results of our study have allowed us to open a debate on possible causes of feeding a child in front of screens, which is not yet well explored. Young children who are the only child in a family are more likely to be placed in front of a screen during meals by their parents. Child’s difficult behaviors can also add to parent’s decision to entertain a child with screens during meals. Thus, health care providers should pay particular attention to parental concerns regarding their children’s eating as especially first-time parents may use screens as tools “for entertainment” during meals with their young children. Parents of young children with emotional and behavioral problems should be provided with additional support and guidance to use consistent and reasonable limits in parenting a child and regulating the screen usage at home and in other children’s environments. Background TV at home was an important predictor of screen use during child’s meals. Therefore, parents should also be educated about the importance of their own screen and eating related behavior in front of their young children. It is extremely important for shaping future health behaviors of their children, especially at the age when no other significant role models are drawing children’s attention. Finally, we highly encourage further longitudinal studies to explore the persistence as well as possible causal effects of screen use during meals on young children’s health.

## 5. Conclusions

In sum, results of our study showed that longer daily screen time, more frequent background TV along with elder child’s age were the main predictors of whether screens were used during child’s meals at least occasionally. In addition, children with longer screen time, with higher exposure to background TV and without siblings were at higher odds to be fed in front of screen daily. Children who are exposed to screens during meals were also reported by their parents as consuming more junk foods and as having more behavioral problems. Thus, in order to prevent children health problems health providers should pay considerable attention to toddlers’ and preschoolers’ screen time and parental behavior regulating screen-based media use. Parents and caretakers of young children should be encouraged to avoid using screens for their children during meals and to introduce clear overall screen time limits. Health care providers should in particular address first-time parents with counselling and supervision regarding screen use during meals.

## Figures and Tables

**Table 1:** Sample characteristics of the participants.

	Variable		N	(%)
Children	Child age, months (mean (SD))	45.50 (13.77)		
	Child BMI (mean (SD))	15.51 (1.91)		
	Child sex			
	Male		467	(51.5)
	Female		410	(48.5)
	Kindergarten attendance			
	Public kindergarten		675	(80.2)
	Private kindergarten		120	(14.3)
	Not attending		47	(5.6)
	Sibling status			
	No siblings		224	(28.1)
	At least one sibling		573	(71.9)
	Daily screen time			
	Less than 1 h		422	(51.6)
	1 h and more		397	(48.4)
Parents	Maternal BMI (mean (SD))	23.05 (4.13)		
	Paternal BMI (mean (SD))	26.22 (3.77)		
	Maternal education			
	Incomplete secondary		13	(1.6)
	Secondary		41	(5.0)
	Secondary vocational		90	(10.9)
	College		115	(13.9)
	University		566	(68.6)
	Paternal education			
	Incomplete secondary		25	(3.3)
	Secondary		56	(7.5)
	Secondary vocational		141	(18.9)
	College		116	(15.5)
	University		410	(54.8)
	Marital status			
	Married		709	(84.3)
	Partnership		85	(10.1)
	Single		41	(4.9)
	Other		6	(0.7)

**Table 2:** Bivariate correlations among variables.

Variable	2	3	4	5	6	7	8	9	10	Child Age
1. Screen use during meals	−0.04	0.34 **	0.16 *	−0.05	−0.05	0.06	0.08 *	0.14 **	0.13 **	0.23 **
2. Child BMI		−0.03	−0.02	−0.08 *	−0.09 *	0.08 *	0.04	0.07	0.05	−0.23 **
3. Daily screen time			0.28 **	−0.14 **	−0.19 **	0.12 **	0.10 *	0.14 **	0.37 **	0.15 **
4. Junk food				0.03	−0.06	0.07 *	0.11 **	0.19 **	0.15 **	0.12 **
5. Maternal education					0.49 **	−0.07 *	−0.06	−0.11 **	−0.22 **	0.04
6. Paternal education						−0.11 **	−0.09 *	−0.15 **	−0.28 **	0.06
7. Maternal BMI							−0.15 **	0.04	0.05	0.05
8. Paternal BMI								−0.01	0.17 **	0.05
9. Total CBCL									0.16 **	−0.06
10. Background TV										−0.07 *

*Note*. * *p* < 0.05; ** *p* < 0.01.

**Table 3:** Multinomial regression analysis representing the independent predictors of screen use during meals.

	Screen Use During Meal					
	*Never ^a^*		*Sometimes ^b^*		*Daily* ^c^	
Variables	OR (95% CI)	*p*	OR (95% CI)	*p*	OR (95% CI)	*p*
Daily screen time	0.99 (0.98–0.99)	<0.001	1.01 (1.00–1.01)	0.006	1.01 (1.00–1.01)	<0.001
Background TV	0.78 (0.66–0.91)	0.002	1.26 (1.10–1.45)	0.001	1.02 (0.87–1.20)	0.810
Total CBCL	0.99 (0.98–1.00)	0.059	1.00 (0.99–1.01)	0.965	1.01 (1.00–1.02)	0.059
No siblings (ref.—at least 1 sibling)	0.42 (0.25–0.69)	0.001	1.41 (0.91–2.19)	0.128	1.70 (1.04–2.79)	0.035
Child age	0.99 (0.97–1.00)	0.131	1.02 (1.00–1.03)	0.022	1.00 (0.98–1.01)	0.694
Paternal BMI	0.97 (0.92–1.03)	0.326	0.99 (0.94–1.04)	0.755	1.04 (0.98–1.10)	0.204
Child BMI	1.08 (0.96–1.22)	0.192	1.03 (0.93–1.13)	0.571	0.90 (0.80–1.01)	0.074
Maternal education	0.86 (0.66–1.10)	0.231	0.98 (0.80–1.21)	0.859	1.19 (0.93–1.52)	0.162

*Note*. OR—Odds Ratio, 95% CI—95% Confidence Interval *^a^*—reference category is “daily”, *^b^*—reference category is “never”, ^c^—reference category is “sometimes”.
